# Acceptance of Lung Cancer Screening and Associated Factors in Hong Kong: A Population‐Based Study

**DOI:** 10.1002/cam4.71513

**Published:** 2026-01-07

**Authors:** Claire Chenwen Zhong, Zhaojun Li, Mingtao Chen, Zehuan Yang, Junjie Huang, Martin C. S. Wong

**Affiliations:** ^1^ JC School of Public Health and Primary Care Faculty of Medicine, The Chinese University of Hong Kong Hong Kong SAR China; ^2^ Centre for Health Education and Health Promotion Faculty of Medicine, The Chinese University of Hong Kong Hong Kong SAR China

**Keywords:** factors, health belief model, LDCT screening, lung cancer, willingness

## Abstract

**Introduction:**

Low‐dose computed tomography (LDCT) enables early detection of lung cancer and reduces mortality, yet public willingness to undergo screening remains suboptimal. This study aimed to assess willingness and its associated factors among high‐risk individuals in Hong Kong.

**Methods:**

A territory‐wide cross‐sectional survey was conducted among adults aged 54 years or above, and those aged 45–54 years with at least one lung cancer risk factor (e.g., smoking, secondhand smoke exposure, or family history) in Hong Kong. Data were collected via self‐administered questionnaires, which included socio‐demographic information, risk exposure, awareness and experience of LDCT, and constructs from the Health Belief Model (HBM). Logistic regression was performed to identify factors associated with willingness to undergo LDCT screening.

**Results:**

A total of 1100 participants were included in the analysis. Among them, 57.3% expressed willingness to undergo LDCT within the next year. Multivariable logistic regression showed that higher self‐efficacy was the strongest factor of willingness, followed by greater perceived benefits and stronger cues to. Additional significant factors included being a current or former smoker, secondhand smoke exposure, age > 65 years, and being responsible for cooking at home. In contrast, unmarried individuals were significantly less likely to be willing to undergo LDCT (aOR = 0.678; 95% CI: 0.486–0.946; *p* = 0.022).

**Conclusion:**

Willingness to undergo LDCT screening was suboptimal among high‐risk individuals in Hong Kong. Key facilitators included higher self‐efficacy, perceived benefits, and cues to action—central domains of the Health Belief Model. Targeted strategies that strengthen these domains may improve screening uptake.

## 1 Introduction

Lung cancer is the leading cause of cancer incidence and mortality worldwide. It has been reported that in 2022, about 2.5 million new lung cancer cases and over 1.8 million deaths were observed worldwide, accounting for one‐eighth and one‐fifth of new cancer cases and deaths among all cancer types, respectively [1]. Predictions indicate that the number of new cases in 40 countries is expected to rise from 1.31 million in 2010 to 2.17 million in 2035, representing a 65.3% increase [2]. Notably, the rate of increase in China (69.7% to 70.0%) exceeds that of the United States (54.5% to 45.5%) by 2050 [3]. The prognosis for lung cancer remains poor, with the majority of diagnoses occurring at advanced stages: 57.3% at stage IV and 15.7% at stage III [4]. In the United States, the latest 5‐year survival rate stands at 20.5%, an improvement from 11.5% in 1975, attributed to earlier detection and advancements in treatment modalities such as immune and targeted therapies [5]. Globally, however, 5‐year survival rates vary significantly among countries, ranging from approximately 10% to 20% in most countries, largely due to ineffective treatment resulting from delayed diagnosis [6, 7]. The World Health Organization (WHO) projects that the lung cancer burden will continue to rise, particularly in the Asia‐Pacific regions [8, 9].

For instance, China is one of the countries with the heaviest burden of lung cancer. Lung cancer burden has continued to grow over the past few decades in China, with an average annual percent change in incidence of 1.15% between 1990 and 2019. Meanwhile, mortality rates and disability‐adjusted life years (DALYs) increased by an average of 0.68% and 0.31% annually, respectively [10]. In Hong Kong, despite a declining trend in incidence (AAPC: −1.4%) and mortality (AAPC: −1.6%) attributable to the implementation of a series of interventions, such as tobacco control, over the past few decades [11, 12], lung cancer continues to be the most prevalent cancer, with 5707 cases reported in 2022, accounting for 16.1% of new cases, particularly affecting males and individuals over 60 years [11]. Lung cancer not only poses health risks but also carries a significant economic burden for patients and the healthcare system. A systematic review of 19 studies highlighted the substantial economic burden of lung cancer, with direct medical costs ranging from US$4484 to US$45,364 (purchasing power parity) [13]. Likewise, Studies from Hong Kong estimated that the lifetime medical expenditures for lung cancer patients range from US$18,455 to US$36,711 [14]. Given this, the Chief Executive's 2024 Policy Address emphasized the need to establish and develop a lung cancer screening strategy to alleviate the disease burden [15].

Screening, as a secondary preventive measure, aims at early detection during the asymptomatic stage [16]. Although emerging evidence suggests that widespread screening may lead to overdiagnosis, especially within the average‐risk population [17], the advantages of lung cancer screening are crucial. A study from the UK indicated that participation in lung cancer screening significantly shifted cancer staging toward earlier phases, with the number of patients diagnosed with stage I or II of lung cancer increasing by 8.8%, while those diagnosed with stage II or IV decreased by 9.3% [18]. Additionally, a prospective cohort study from China involving approximately 1 million participants found that early screening led to a 31.0% reduction in lung cancer mortality and a 32.0% decrease in all‐cause mortality [19]. Similarly, a study conducted in Taiwan reported analogous findings, emphasizing that the implementation of lung cancer screening significantly decreased lung cancer mortality and modified the distribution of lung cancer stages [20]. This evidence further reveals the importance of early diagnosis of lung cancer and early screening in alleviating the burden of lung cancer.

Low‐dose computed tomography (LDCT) is a widely used strategy for lung cancer screening with high sensitivity for detecting lung cancer in high‐risk individuals [21]. Many countries, such as the United States [22] Korea [23], and China [24], recommend screening for lung cancer in high‐risk populations using LDCT for preventive intervention. However, the participation rate in LDCT‐based lung cancer screening is suboptimal, the prevalence estimated to be only 16.4%–21.3% in the United States and about 36.6% in Korea among the eligible population [25, 26, 27].

Previous research on individuals' willingness to undergo lung cancer screening has shown that health beliefs and characteristics play an important role in their health behavior decision [28]. With this in mind, the Health Belief Model (HBM) has been used extensively to gain insight into the role of perceived benefits, barriers, susceptibility, severity, self‐efficacy, and cues to action on behavioral decisions among individuals from various contexts [29]. The linkage between HBM‐related factors and the LDCT screening behavior has been well‐identified [30]. Perceived benefits, such as enhanced treatment outcomes, may motivate individuals to undergo LDCT screening. Conversely, individuals experiencing fear, financial constraints, or insufficient information tend to exhibit greater resistance and hold negative attitudes toward lung cancer screening [30].

However, to the best of our knowledge, although extensive research has explored lung cancer trends and risk factors in Hong Kong, there remains a lack of targeted studies to uncover the perspectives and willingness of potential service recipients of lung cancer screening services to assist the formulation and refinement of local health policies and interventions. Therefore, this study aims to (1) assess the willingness to undergo LDCT‐based lung cancer screening among the high‐risk population and (2) explore the factors associated with the willingness to provide information and assist in the development of targeted screening initiatives.

## 2 Materials and Method

### 2.1 Study Design and Participants

This is a territory‐wide cross‐sectional study to investigate the factors associated with willingness to undergo LDCT among adults in Hong Kong. The study was designed and reported following the Strengthening the Reporting of Observational Studies in Epidemiology Statement [31]. Convenience sampling was adopted to reach potential participants and were recruited through Qualtrics' questionnaire dashboard and online advertisement. Eligible participants in this study were individuals aged above 54 years, as well as those aged 45–54 years with at least one of the following risk conditions: (1) Suffering from chronic obstructive pulmonary disease; (2) Family history of lung cancer; (3) Employment in industries with long‐term dust exposure; (4) Smoking; (5) Exposure to secondhand smoke at home or working environment; (6) Primary responsible for cooking at home. The selection of risk factors is based on evidence from the literature, balancing well‐established factors with region‐specific or emerging factors like cooking fumes [32, 33]. The age threshold was determined by referencing existing screening recommendations and considering the trend of early‐onset lung cancer in Hong Kong. Currently, international organizations emphasize the need for screening among the population starting at aged 55 and above [34, 35]. Furthermore, recent studies and the Hong Kong Cancer Registry indicate an increasing trend of prevalence among the younger age group and a resurgence in the incidence of lung cancer within the 45–54 age group in recent years, warranting caution [36, 37]. Establishing this threshold enables a more thorough exploration of perspectives across age groups and the feasibility of lowering the screening age to inform policy development. As the willingness of lung cancer screening of LDCT had not been systematically evaluated before, we assumed that 50% of participants would report an interested outcome in this study, and therefore at least 1068 valid questionnaires were required to achieve a precision of ±3% and 5% Types I error rate based on the formula: [*N* = 1.96^2^ × *p*(1 − *p*)/precision^2^] [38].

### 2.2 Data Collection

Data were collected via an electronic questionnaire platform, Qualtrics. A pilot test involving a total of 36 targeted participants was conducted to verify the readability and relevance of the questionnaire prior to data collection. As no ambiguous or irrelevant words or sentences were found, no adjustments were made to the questionnaire after the pilot test. All participants were required to complete a self‐administered questionnaire on the Qualtrics platform to collect data on their socio‐demographic characteristics, willingness to undergo LDCT, and view of LDCT. The questionnaire took approximately 10–20 min to complete. Prior to filling out the questionnaire, all participants were informed about the voluntary nature and anonymity of this study, and electronic informed consent was collected before participation. All data were anonymized to ensure that no personally identifiable information could be tracked or available. This study was approved by the Survey and Behavioral Research Ethics Committee of the Chinese University of Hong Kong (SBRE‐24‐0083).

### 2.3 Measurements

A structured questionnaire was developed to capture information across multiple domains, including sociodemographic background, health‐related characteristics, and psychosocial beliefs and attitudes related to lung cancer screening. The questionnaire items were adapted from validated instruments, including the Lung Cancer Screening Health Belief Scales [39], to ensure content validity and conceptual consistency.

Central to the measurement strategy were the HBM model, which assessed six key domains hypothesized to be associated with screening behavior. These included perceived susceptibility to lung cancer (3 items); perceived severity of the disease (5 items); perceived benefits of lung cancer screening (6 items); perceived barriers to undergoing LDCT (17 items); self‐efficacy in completing the screening (9 items); and cues to action that might prompt screening behavior (10 items). Each domain was measured using multiple items rated on a 4‐point Likert scale ranging from “strongly disagree” (score: 1) to “strongly agree” (score: 4), with higher scores reflecting greater endorsement of each domain. For analytical purposes, the total score of each domain was computed, and participants were dichotomized into “high” and “low” levels based on the median value of the distribution. A composite variable for perceived threat of lung cancer was also created by summing the scores for perceived susceptibility and severity, which was similarly categorized into high and low perceived threat based on the median. The scale of the HBM‐construct used in this study shows good performance, with Cronbach's alpha ranging from 0.85 to 0.91 across domains.

In addition to HBM‐related questions, the questionnaire captured key sociodemographic characteristics, including age (categorized as 45–54, 55–65, and over 65 years), sex, marital status, education level (secondary or below, post‐secondary sub‐degree, post‐secondary degree or above), employment status (full‐time or not), and monthly household income (categorized as less than HKD 30,000; HKD 30,000–49,999; and HKD 50,000 or more). Health‐related information was also collected through self‐report and included self‐rated health status (categorized as good vs. not good), presence of chronic disease, smoking status (never smoker vs. current or former smoker), exposure to secondhand smoke (never, seldom, or frequently), frequency of home cooking, long‐term environmental exposure to dust or fumes, family history of lung cancer, and type of health insurance coverage (government‐funded, private, or both).

### 2.4 Outcomes

The study assessed three key outcomes related to participants' engagement with lung cancer screening. The primary outcome was willingness to undergo LDCT screening within the next 12 months. Participants were asked to rate their willingness on a 4‐point Likert scale, ranging from “not willing at all” to “very willing.” For statistical analysis, responses were dichotomized into “willing” (agree or strongly agree to undergo screening) and “not willing” (disagree or strongly disagree).

The secondary outcomes included participants' awareness of LDCT and their prior experience with LDCT screening. Awareness was assessed through a single binary item that asked whether participants had ever heard of low‐dose computed tomography as a lung cancer screening method. Experience was similarly measured by asking whether participants had ever undergone an LDCT scan. These measures were selected to capture both behavioral intention and actual behavior related to lung cancer screening and to examine how these outcomes were associated with HBM‐related beliefs as well as sociodemographic and health‐related factors.

### 2.5 Statistical Analysis

All statistical analyses were conducted using R version 4.2.1. Descriptive statistics, including frequencies and percentages, were first computed to summarize the characteristics of the study population. To examine differences in sociodemographic characteristics, health‐related factors, and HBM constructs between participants who were willing versus not willing to undergo LDCT screening within the next 12 months, chi‐square tests were performed for categorical variables. To identify factors associated with the three outcome variables—willingness to undergo LDCT screening, awareness of LDCT, and previous LDCT experience—logistic regression analyses were conducted. For each outcome, univariable logistic regression models were first estimated to examine the crude association between each independent variable and the outcome of interest. Multivariable models were conducted incorporating a comprehensive set of independent variables, including sociodemographic factors (age, sex, marital status, education level, employment status, and household income), health‐related characteristics (self‐reported health status, presence of chronic diseases, smoking status, secondhand smoke exposure, environmental exposure, cooking frequency, family history of lung cancer, and health insurance coverage), and psychological factors grounded in the HBM (perceived threat of lung cancer, perceived benefits and barriers of LDCT, self‐efficacy, and cues to action). The crude odds ratios (OR), adjusted odds ratios (aOR) and their 95% confidence intervals (CI) were calculated for univariable and multivariable models respectively. The Variance Inflation Factor (VIF) test was used to assess multicollinearity among variables. Variables with generalized VIF (GVIF) > 5 were excluded in the multivariable logistic model [40]. Model fit was assessed using the Hosmer–Lemeshow goodness‐of‐fit test, with non‐significant *p*‐values indicating adequate fit. A two‐tailed *p*‐value of < 0.05 was considered statistically significant.

## 3 Results

### 3.1 Participant's Characteristic

A total of 1100 participants were included in this study, comprising 535 (48.6%) males and 565 (51.4%) females. Of these, 57.3% (*n* = 630) of participants expressed willingness to undergo LDCT within 1 year, while 42.7% (*n* = 470) were opposed. The largest age group was 45–54 years, accounting for 560 participants (50.9%). Regarding environmental and lifestyle risk factors, 183 participants (16.6%) reported long‐term exposure to high‐risk environments, and 409 (37.2%) were current or former smokers. Additionally, 753 participants (68.5%) identified themselves as the primary cook in their household, and only 248 (22.5%) reported never exposure to secondhand smoke. Details could be found in the Table 1.

**TABLE 1:** Participant characteristics by willingness to receive low‐dose CT within 1 year.

Variable	Overall	Disagree	Agree	*p*
Number	1100	470 (42.7)	630 (57.3)	
Age group				0.950
45–54 years	560 (50.9)	237 (42.3)	323 (57.7)	
55–65 years	448 (40.7)	194 (43.3)	254 (56.7)	
Over 65 years	92 (8.4)	39 (42.4)	53 (57.6)	
Sex				**0.001**
Male	535 (48.6)	200 (37.4)	335 (62.6)	
Female	565 (51.4)	270 (47.8)	295 (52.2)	
Marriage				**< 0.001**
Married	792 (72.0)	295 (37.2)	497 (62.8)	
Unmarried	308 (28.0)	175 (56.8)	133 (43.2)	
Education				**0.007**
Secondary school or below	408 (37.1)	190 (46.6)	218 (53.4)	
Post‐secondary sub‐degree	207 (18.8)	69 (33.3)	138 (66.7)	
Post‐secondary degree or above	485 (44.1)	211 (43.5)	274 (56.5)	
Employment				**0.010**
Full‐time	810 (73.6)	327 (40.4)	483 (59.6)	
Non‐full time	290 (26.4)	143 (49.3)	147 (50.7)	
Household income (Monthly)				**< 0.001**
	308 (28.0)	159 (51.6)	149 (48.4)	
30,000‐49,999	445 (40.5)	159 (35.7)	286 (64.3)	
≥ 50,000	347 (31.5)	152 (43.8)	195 (56.2)	
Self‐report health				0.675
Good	496 (45.1)	208 (41.9)	288 (58.1)	
Not Good	604 (54.9)	262 (43.4)	342 (56.6)	
Health insurance				**0.001**
No	421 (38.3)	207 (49.2)	214 (50.8)	
Yes	679 (61.7)	263 (38.7)	416 (61.3)	
Chronic disease				**0.014**
No	523 (47.5)	244 (46.7)	279 (53.3)	
Yes	577 (52.5)	226 (39.2)	351 (60.8)	
Risky environment				**< 0.001**
No	917 (83.4)	422 (46.0)	495 (54.0)	
Yes	183 (16.6)	48 (26.2)	135 (73.8)	
Smoking				**< 0.001**
Never smoker	691 (62.8)	353 (51.1)	338 (48.9)	
Current/ex‐smoker	409 (37.2)	117 (28.6)	292 (71.4)	
Secondhand smoke				**< 0.001**
Never	248 (22.5)	135 (54.4)	113 (45.6)	
Seldom	687 (62.5)	298 (43.4)	389 (56.6)	
Frequently	165 (15.0)	37 (22.4)	128 (77.6)	
Cooking at home				**0.012**
No	347 (31.5)	168 (48.4)	179 (51.6)	
Yes	753 (68.5)	302 (40.1)	451 (59.9)	
Family history of lung cancer				**< 0.001**
No	913 (83.0)	414 (45.3)	499 (54.7)	
Yes	187 (17.0)	56 (29.9)	131 (70.1)	
Perceived threat				**< 0.001**
Low	614 (55.8)	312 (50.8)	302 (49.2)	
High	486 (44.2)	158 (32.5)	328 (67.5)	
Perceived benefits				**< 0.001**
Low	610 (55.5)	330 (54.1)	280 (45.9)	
High	490 (44.5)	140 (28.6)	350 (71.4)	
Perceived barriers				**0.048**
Low	607 (55.2)	276 (45.5)	331 (54.5)	
High	493 (44.8)	194 (39.3)	299 (60.7)	
Self‐efficacy				**< 0.001**
Low	565 (51.4)	338 (59.8)	227 (40.2)	
High	535 (48.6)	132 (24.7)	403 (75.3)	
Cues to action				**< 0.001**
Low	554 (50.4)	304 (54.9)	250 (45.1)	
High	546 (49.6)	166 (30.4)	380 (69.6)	

*Note:* Disagree: Participants disagree or strongly disagree with “I plan to accept Low‐dose CT within 1 year”; Agree: Participants agree or strongly agree with “I plan to accept Low‐dose CT within 1 year”; Risky environment: Participants had long‐term exposure to dust or cooking fume environment; Low & High: The perceived threat, perceived benefits, perceived barriers, self‐efficacy, and cues to action scales use 4‐point Likert‐style responses from strongly agree to strongly disagree, and were divided into low and high groups by their median points. Significant values are in bold.

Chi‐square analyses revealed statistically significant differences in willingness to undergo LDCT across various sociodemographic, behavioral, and psychosocial factors (Table 1). A significantly higher proportion of participants who were male (*p* = 0.001), married (*p* < 0.001), employed full‐time (*p* = 0.010), had health insurance (*p* = 0.001), suffered from chronic disease (*p* = 0.014), had long‐term exposure to risky environments (*p* < 0.001), were current or former smokers (*p* < 0.001), primarily cooked at home (*p* = 0.012), or had a family history of lung cancer (*p* < 0.001) expressed willingness to undergo LDCT compared to their respective counterparts. In addition, significant differences in willingness were observed across participants with different levels of household income (*p* < 0.001), education (*p* < 0.001), and frequency of secondhand smoke exposure (*p* < 0.001). Regarding HBM domains, willingness to undergo LDCT differed significantly by levels of perceived threat, perceived benefits, self‐efficacy, and cues to action (all *p* < 0.001), with higher scores on these constructs being associated with greater willingness to undergo screening.

### 3.2 Result of Logistic Regression Among Outcomes

Tables 2, 3, 4 present factors associated with willingness to undergo LDCT, awareness of LDCT, and the LDCT experience, respectively. The key findings on the factor association are summarized in Table S1 and Figure S1. No variables exhibited multicollinearity in the VIF test (Table S2).

**TABLE 2:** Factors associated with willingness to receive low‐dose CT within 1 year.

Variable	Number of participant	OR	*p*	Adjusted_OR	*p*
Age group					
45–54 years	560 (50.9)	Reference		Reference	
55–65 years	448 (40.7)	0.961 (0.747–1.235)	0.754	1.081 (0.795–1.469)	0.620
Over 65 years	92 (8.4)	0.997 (0.638–1.558)	0.990	1.900 (1.074–3.362)	**0.027**
Sex					
Male	535 (48.6)	Reference		Reference	
Female	565 (51.4)	0.652 (0.513–0.830)	**0.001**	0.935 (0.689–1.269)	0.667
Marriage					
Married	792 (72.0)	Reference		Reference	
Unmarried	308 (28.0)	0.451 (0.345–0.589)	**< 0.001**	0.678 (0.486–0.946)	**0.022**
Education					
Secondary school or below	408 (37.1)	Reference		Reference	
Post‐secondary sub‐degree	207 (18.8)	1.743 (1.230–2.470)	**0.002**	1.438 (0.957–2.162)	0.081
Post‐secondary degree or above	485 (44.1)	1.132 (0.869–1.475)	0.359	0.864 (0.619–1.208)	0.394
Employment					
Full‐time	810 (73.6)	Reference		Reference	
Non‐full time	290 (26.4)	0.696 (0.531–0.911)	**0.008**	0.878 (0.617–1.248)	0.467
Household income (monthly)					
	308 (28.0)	Reference		Reference	
30,000‐49,999	445 (40.5)	1.919 (1.428–2.580)	**< 0.001**	1.210 (0.836–1.750)	0.312
≥ 50,000	347 (31.5)	1.369 (1.006–1.863)	**0.046**	0.838 (0.557–1.261)	0.396
Self‐report health					
Good	496 (45.1)	Reference		Reference	
Not Good	604 (54.9)	0.943 (0.741–1.199)	0.630	1.153 (0.848–1.566)	0.364
Health insurance					
No	421 (38.3)	Reference		Reference	
Yes	679 (61.7)	1.530 (1.197–1.956)	**0.001**	1.227 (0.908–1.659)	0.183
Chronic disease					
No	523 (47.5)	Reference		Reference	
Yes	577 (52.5)	1.358 (1.069–1.726)	**0.012**	0.939 (0.691–1.276)	0.687
Risky environment					
No	917 (83.4)	Reference		Reference	
Yes	183 (16.6)	2.398 (1.683–3.416)	**< 0.001**	1.418 (0.939–2.141)	0.097
Smoking					
Never smoker	691 (62.8)	Reference		Reference	
Current/ex‐smoker	409 (37.2)	2.606 (2.007–3.385)	**< 0.001**	1.710 (1.220–2.396)	**0.002**
Secondhand smoke					
Never	248 (22.5)	Reference		Reference	
Seldom	687 (62.5)	1.560 (1.165–2.088)	**0.003**	1.380 (0.974–1.955)	0.070
Frequently	165 (15.0)	4.133 (2.654–6.437)	**< 0.001**	2.099 (1.236–3.566)	**0.006**
Cooking at home					
No	347 (31.5)	Reference		Reference	
Yes	753 (68.5)	1.402 (1.085–1.811)	**0.010**	1.476 (1.082–2.013)	**0.014**
Family history of lung cancer					
No	913 (83.0)	Reference		Reference	
Yes	187 (17.0)	1.941 (1.383–2.724)	**< 0.001**	1.442 (0.963–2.160)	0.076
Perceived threat					
Low	614 (55.8)	Reference		Reference	
High	486 (44.2)	2.145 (1.675–2.746)	**< 0.001**	1.191 (0.876–1.619)	0.265
Perceived benefits					
Low	610 (55.5)	Reference		Reference	
High	490 (44.5)	2.946 (2.289–3.793)	**< 0.001**	1.921 (1.428–2.586)	**< 0.001**
Perceived barriers					
Low	607 (55.2)	Reference		Reference	
High	493 (44.8)	2.115 (1.657–2.700)	**< 0.001**	1.263 (0.944–1.689)	0.116
Self‐efficacy					
Low	565 (51.4)	Reference		Reference	
High	535 (48.6)	4.546 (3.510–5.888)	**< 0.001**	3.313 (2.461–4.462)	**< 0.001**
Cues to action					
Low	554 (50.4)	Reference		Reference	
High	546 (49.6)	2.784 (2.173–3.565)	**< 0.001**	1.672 (1.252–2.231)	**< 0.001**

*Note:* Risky environment: Participants had long‐term exposure to dust or cooking fume environment; Low & High: The perceived threat, perceived benefits, perceived barriers, self‐efficacy, and cues to action scales use 4‐point Likert‐style responses from strongly agree to strongly disagree, and were divided into low and high groups by their median points. *χ* ^2^ = 11.586, df = 8, *p*‐value = 0.171. Significant values are in bold.

#### 3.2.1 Factors Associated With Willingness to Receive LDCT

Univariable analyses identified 17 factors associated with willingness to undergo LDCT (Table 2). After adjusting for covariates, eight factors remained statistically significant. Self‐efficacy exhibited the strongest positive association; participants with high self‐efficacy had more than threefold higher odds of willingness compared to those with low self‐efficacy (aOR = 3.313; 95% CI: 2.461–4.462; *p* < 0.001). Higher perceived benefits of LDCT (aOR = 1.921; 95% CI: 1.428–2.586; *p* < 0.001) and stronger cues to action (aOR = 1.672; 95% CI: 1.252–2.231; *p* < 0.001) were also positively associated with willingness. Participants who were current or former smokers had significantly higher odds of willingness compared to non‐smokers (aOR = 1.710; 95% CI: 1.220–2.396; *p* = 0.002), as did those frequently exposed to secondhand smoke (aOR = 2.099; 95% CI: 1.236–3.566; *p* = 0.006), individuals aged over 65 years (aOR = 1.900; 95% CI: 1.074–3.362; *p* = 0.027), and those primarily responsible for cooking at home (aOR = 1.476; 95% CI: 1.082–2.013; *p* = 0.014). Conversely, unmarried status was negatively associated with willingness (aOR = 0.678; 95% CI: 0.486–0.946; *p* = 0.022).

#### 3.2.2 Factors Associated With Awareness of LDCT

Table 3 presents factors associated with awareness of LDCT screening. Although multiple factors were significant in univariable analyses, the multivariable logistic regression results are emphasized here. Unmarried participants also had significantly lower odds of awareness of LDCT compared to married individuals (aOR = 0.602; 95% CI: 0.403–0.897; *p* = 0.013). Similarly, participants with a monthly household income of HKD 50,000 or above had reduced odds of awareness relative to those earning less than HKD 30,000 (aOR = 0.491; 95% CI: 0.305–0.792; *p* = 0.004). Conversely, having health insurance was associated with a 77% increase in odds of awareness (aOR = 1.767; 95% CI: 1.253–2.492; *p* = 0.001). Current or former smokers (aOR = 1.907; 95% CI: 1.336–2.723; *p* < 0.001) and those with a family history of lung cancer (aOR = 1.962; 95% CI: 1.329–2.896; *p* = 0.001) were also significantly more likely to be aware of LDCT. Regarding the Health Belief Model constructs, cues to action emerged as the strongest predictor of awareness (aOR = 2.631; 95% CI: 1.894–3.653; *p* < 0.001), followed by self‐efficacy (aOR = 1.705; 95% CI: 1.224–2.374; *p* = 0.002) and perceived benefits (aOR = 1.680; 95% CI: 1.218–2.316; *p* = 0.002).

**TABLE 3:** Factors associated with awareness of low‐dose CT.

Variable	Number of participant	OR	*p*	Adjusted_OR	*p*
Age group					
45–54 years	560 (50.9)	Reference		Reference	
55–65 years	448 (40.7)	0.873 (0.662–1.153)	0.339	0.900 (0.648–1.248)	0.527
Over 65 years	92 (8.4)	0.328 (0.170–0.632)	**0.001**	0.504 (0.237–1.073)	0.075
Sex					
Male	535 (48.6)	Reference		Reference	
Female	565 (51.4)	0.862 (0.660–1.126)	0.275	1.317 (0.945–1.835)	0.104
Marriage					
Married	792 (72.0)	Reference		Reference	
Unmarried	308 (28.0)	0.448 (0.320–0.628)	**< 0.001**	0.602 (0.403–0.897)	**0.013**
Education					
Secondary school or below	408 (37.1)	Reference		Reference	
Post‐secondary sub‐degree	207 (18.8)	1.186 (0.811–1.734)	0.379	0.874 (0.564–1.353)	0.545
Post‐secondary degree or above	485 (44.1)	1.241 (0.919–1.676)	0.158	1.064 (0.736–1.538)	0.742
Employment					
Full‐time	810 (73.6)	Reference		Reference	
Non‐full time	290 (26.4)	0.714 (0.520–0.980)	**0.037**	1.057 (0.717–1.559)	0.779
Household income (monthly)					
	308 (28.0)	Reference		Reference	
30,000‐49,999	445 (40.5)	1.827 (1.306–2.557)	**< 0.001**	0.939 (0.630–1.400)	0.758
≥ 50,000	347 (31.5)	1.099 (0.759–1.590)	0.618	0.491 (0.305–0.792)	**0.004**
Self‐report health					
Good	496 (45.1)	Reference		Reference	
Not Good	604 (54.9)	0.808 (0.618–1.056)	0.118	0.876 (0.626–1.227)	0.441
Health insurance					
No	421 (38.3)	Reference		Reference	
Yes	679 (61.7)	2.196 (1.632–2.956)	**< 0.001**	1.767 (1.253–2.492)	**0.001**
Chronic disease					
No	523 (47.5)	Reference		Reference	
Yes	577 (52.5)	1.450 (1.106–1.900)	**0.007**	1.293 (0.923–1.810)	0.135
Risky environment					
No	917 (83.4)	Reference		Reference	
Yes	183 (16.6)	2.031 (1.456–2.833)	**< 0.001**	1.185 (0.799–1.758)	0.399
Smoking					
Never smoker	691 (62.8)	Reference		Reference	
Current/ex‐smoker	409 (37.2)	2.724 (2.071–3.582)	**< 0.001**	1.907 (1.336–2.723)	**< 0.001**
Secondhand smoke					
Never	248 (22.5)	Reference		Reference	
Seldom	687 (62.5)	1.392 (0.972–1.993)	0.071	1.206 (0.802–1.815)	0.368
Frequently	165 (15.0)	3.388 (2.183–5.260)	**< 0.001**	1.634 (0.965–2.768)	0.068
Cooking at home					
No	347 (31.5)	Reference		Reference	
Yes	753 (68.5)	1.184 (0.884–1.586)	0.257	0.991 (0.708–1.389)	0.960
Family history of lung cancer					
No	913 (83.0)	Reference		Reference	
Yes	187 (17.0)	2.309 (1.663–3.206)	**< 0.001**	1.962 (1.329–2.896)	**0.001**
Perceived threat					
Low	614 (55.8)	Reference		Reference	
High	486 (44.2)	1.758 (1.343–2.301)	**< 0.001**	0.843 (0.602–1.181)	0.320
Perceived benefits					
Low	610 (55.5)	Reference		Reference	
High	490 (44.5)	2.430 (1.848–3.194)	**< 0.001**	1.680 (1.218–2.316)	**0.002**
Perceived barriers					
Low	607 (55.2)	Reference		Reference	
High	493 (44.8)	1.145 (0.876–1.497)	0.322	0.927 (0.674–1.273)	0.638
Self‐efficacy					
Low	565 (51.4)	Reference		Reference	
High	535 (48.6)	2.755 (2.082–3.645)	**< 0.001**	1.705 (1.224–2.374)	**0.002**
Cues to action					
Low	554 (50.4)	Reference		Reference	
High	546 (49.6)	3.994 (2.975–5.362)	**< 0.001**	2.631 (1.894–3.653)	**< 0.001**

*Note:* Risky environment: Participants had long‐term exposure to dust or cooking fume environment; Low & High: The perceived threat, perceived benefits, perceived barriers, self‐efficacy, and cues to action scales use 4‐point Likert‐style responses from strongly agree to strongly disagree, and were divided into low and high groups by their median points. *χ* ^2^ = 8.3227, df = 8, *p*‐value = 0.403. Significant values are in bold.

#### 3.2.3 Factors Associated With LDCT Experience

Table 4 details factors associated with having undergone LDCT screening. Of the 16 significant factors identified in univariable analyses, the multivariable model highlighted that participants with health insurance were significantly more likely to have experience with LDCT (aOR = 2.538; 95% CI: 1.441–4.471; *p* = 0.001). Current or former smokers had twice the odds of LDCT experience compared to non‐smokers (aOR = 2.003; 95% CI: 1.178–3.403; *p* = 0.010). Similarly, participants with a family history of lung cancer were more than twice as likely to have undergone LDCT (aOR = 2.664; 95% CI: 1.583–4.483; *p* < 0.001). Cues to action again showed the strongest positive association (aOR = 3.859; 95% CI: 2.169–6.863; *p* < 0.001). Interestingly, higher perceived barriers to LDCT were associated with increased odds of having undergone the screening (aOR = 1.831; 95% CI: 1.151–2.913; *p* = 0.011), potentially reflecting heightened perceived need or physician recommendation despite obstacles. Notably, participants rating their health as poor had significantly lower odds of LDCT experience (aOR = 0.506; 95% CI: 0.307–0.833; *p* = 0.007), indicating potential barriers in this subgroup.

**TABLE 4:** Factors associated with having received low‐dose CT.

Variable	Number of participant	OR	*p*	Adjusted_OR	*p*
Age group					
45–54 years	560 (50.9)	Reference		Reference	
55–65 years	448 (40.7)	0.848 (0.572–1.258)	0.413	0.838 (0.515–1.363)	0.476
Over 65 years	92 (8.4)	0.244 (0.075–0.792)	**0.019**	0.434 (0.118–1.598)	0.210
Sex					
Male	535 (48.6)	Reference		Reference	
Female	565 (51.4)	0.570 (0.386–0.842)	**0.005**	0.858 (0.533–1.381)	0.528
Marriage					
Married	792 (72.0)	Reference		Reference	
Unmarried	308 (28.0)	0.558 (0.344–0.904)	**0.018**	0.993 (0.544–1.812)	0.981
Education					
Secondary school or below	408 (37.1)	Reference		Reference	
Post‐secondary sub‐degree	207 (18.8)	1.158 (0.665–2.015)	0.604	0.724 (0.381–1.377)	0.325
Post‐secondary degree or above	485 (44.1)	1.323 (0.859–2.037)	0.205	0.911 (0.527–1.573)	0.737
Employment					
Full‐time	810 (73.6)	Reference		Reference	
Non‐full time	290 (26.4)	0.769 (0.486–1.215)	0.260	1.560 (0.885–2.751)	0.124
Household income (monthly)					
	308 (28.0)	Reference		Reference	
30,000‐49,999	445 (40.5)	1.968 (1.190–3.257)	**0.008**	1.139 (0.628–2.068)	0.668
≥ 50,000	347 (31.5)	1.346 (0.774–2.340)	0.292	0.662 (0.320–1.369)	0.266
Self‐report health					
Good	496 (45.1)	Reference		Reference	
Not Good	604 (54.9)	0.525 (0.356–0.775)	**0.001**	0.506 (0.307–0.833)	**0.007**
Health insurance					
No	421 (38.3)	Reference		Reference	
Yes	679 (61.7)	3.382 (2.057–5.562)	**< 0.001**	2.538 (1.441–4.471)	**0.001**
Chronic disease					
No	523 (47.5)	Reference		Reference	
Yes	577 (52.5)	1.810 (1.215–2.698)	**0.004**	1.560 (0.943–2.581)	0.084
Risky environment					
No	917 (83.4)	Reference		Reference	
Yes	183 (16.6)	3.009 (1.976–4.581)	**< 0.001**	1.631 (0.976–2.724)	0.062
Smoking					
Never smoker	691 (62.8)	Reference		Reference	
Current/ex‐smoker	409 (37.2)	3.835 (2.562–5.741)	**< 0.001**	2.003 (1.178–3.403)	**0.010**
Secondhand smoke					
Never	248 (22.5)	Reference		Reference	
Seldom	687 (62.5)	1.747 (0.962–3.172)	0.067	1.328 (0.681–2.588)	0.405
Frequently	165 (15.0)	5.173 (2.706–9.889)	**< 0.001**	1.979 (0.910–4.304)	0.085
Cooking at home					
No	347 (31.5)	Reference		Reference	
Yes	753 (68.5)	1.547 (0.991–2.413)	0.055	1.203 (0.727–1.990)	0.471
Family history of lung cancer					
No	913 (83.0)	Reference		Reference	
Yes	187 (17.0)	3.489 (2.308–5.273)	**< 0.001**	2.664 (1.583–4.483)	**< 0.001**
Perceived threat					
Low	614 (55.8)	Reference		Reference	
High	486 (44.2)	2.147 (1.452–3.175)	**< 0.001**	0.792 (0.478–1.312)	0.365
Perceived benefits					
Low	610 (55.5)	Reference		Reference	
High	490 (44.5)	2.288 (1.543–3.393)	**< 0.001**	1.287 (0.798–2.076)	0.301
Perceived barriers					
Low	607 (55.2)	Reference		Reference	
High	493 (44.8)	2.353 (1.584–3.496)	**< 0.001**	1.831 (1.151–2.913)	**0.011**
Self‐efficacy					
Low	565 (51.4)	Reference		Reference	
High	535 (48.6)	2.781 (1.837–4.209)	**< 0.001**	1.464 (0.885–2.420)	0.138
Cues to action					
Low	554 (50.4)	Reference		Reference	
High	546 (49.6)	7.169 (4.224–12.168)	**< 0.001**	3.859 (2.169–6.863)	**< 0.001**

*Note:* Risky environment: Participants had long‐term exposure to dust or cooking fume environment; Low & High: The perceived threat, perceived benefits, perceived barriers, self‐efficacy, and cues to action scales use 4‐point Likert‐style responses from strongly agree to strongly disagree, and were divided into low and high groups by their median points. χ^2^ = 8.0079, df = 8, *p*‐value = 0.433. Significant values are in bold.

## 4 Discussion

### 4.1 Major Findings

This cross‐sectional study examined willingness to undergo LDCT screening and its associated factors among high‐risk individuals for lung cancer in Hong Kong. Results showed that willingness to undergo LDCT was suboptimal, with only 57.3% of participants expressing willingness to receive screening within the next year. Multivariable analysis identified that higher self‐efficacy showed the strongest positive association, followed by higher perceived benefits, stronger cues to action, smoking status, secondhand smoke exposure, older age, and being responsible for cooking at home. In contrast, unmarried individuals were significantly less willing to undergo LDCT. On the other hand, awareness of LDCT screening was positively associated with having health insurance, smoking status, family history of lung cancer, and higher levels of cues to action, self‐efficacy, and perceived benefits. However, unmarried participants and those with higher household incomes were less likely to be aware of LDCT. Actual experience with LDCT screening was associated with cues to action, family history of lung cancer, smoking status, health insurance, and perceived barriers. Participants who rated their health as poor were significantly less likely to have undergone LDCT.

### 4.2 Evidence From Prior Studies

Our study revealed a suboptimal level (57.3%) of willingness to receive LDCT among high‐risk populations in Hong Kong. This is consistent with a study in mainland China, where only 58.12% of high‐risk individuals reported willingness to undergo lung cancer screening [41]. A 2023 cross‐sectional study in Korea found a moderate level of intention, with a mean score of 3.66 out of 5 among 186 high‐risk individuals [25]. In contrast, much higher levels of willingness were reported in Western countries. For example, 84.3% of high‐risk individuals in Belgium and 86% in the United States expressed willingness to participate in lung cancer screening [42, 43]. These findings suggest that willingness to undergo LDCT screening among high‐risk populations in East Asian countries remains relatively low and highlights the need for targeted strategies to improve screening uptake and reduce the burden of lung cancer in the region.

Multiple factors have been identified as associated with risk groups' attitudes toward LDCT screening. Among these, HBM‐related domains such as self‐efficacy, cues to action, and risk exposure show the strongest association with perceptions of LDCT screening, underscoring their importance in shaping public health strategies. Understanding these factors that influence people's willingness to undergo screening can help develop targeted interventions to improve screening willingness and awareness among high‐risk groups.

Self‐efficacy was identified as having the strongest association with the LDCT willingness and awareness in this study, which aligns with previous research. A Korean study also found self‐efficacy to be a significant predictor of lung cancer screening intention [25] Two US studies also reported that higher self‐efficacy scores were positively associated with greater intention to undergo lung cancer screening or actual participation [44, 45]. It has been suggested that individuals with higher self‐efficacy tend to have higher incomes, a family history of lung cancer, lower fear of screening, and reduced belief in cancer fatalism [44] They are also more likely to receive screening recommendations and have stronger social support networks, which may further enhance their confidence and motivation to participate in screening [44].

Perceived benefits were also significantly associated with willingness and awareness of LDCT screening, consistent with findings from previous studies [25, 44, 46, 47, 48]. A qualitative study found that low perceived benefit of lung cancer screening was associated with declining participation rates, particularly the belief that early cancer detection offered negligible advantages in advanced age [49]. In addition, cues to action were significantly associated with a positive perception of LDCT. Studies across different populations have identified several cues that motivate screening for high‐risk populations [46, 50, 51]. One study categorized these cues into four key domains, including family encouragement, recommendations from healthcare professionals, exposure to educational materials, and presence of symptoms [51]. Likewise, this may account for the observation that a married individual might exhibit a more favorable perspective of LDCT compared to their counterpart. This could be attributed to cues from family or social role perceptions that promote engagement in healthy behaviors [52]. These findings highlight the importance of targeted interventions that strengthen self‐efficacy, emphasize the benefits of early detection, and activate appropriate cues to action to increase willingness and perceptions of participation in LDCT screening.

Individual with risk exposure, such as smoking and secondhand smoke, demonstrates strong willingness to undergo LDCT screening, exhibits higher awareness, and is more likely to have previously undergone LDCT screening. A 2021 cross‐sectional study of over 35,000 Chinese residents found that smoking was positively correlated with awareness that LDCT can detect early‐stage lung cancer [53]. Likewise, a cross‐sectional study among smokers reported that participants who were moderately or very concerned about smoking‐related health risks had 20% higher odds of intending to undergo LDCT compared to those with low concern, highlighting the role of perceived harm in motivating screening behavior [43].

However, a concerning phenomenon is that despite smokers having a higher willingness to participate in lung cancer screening, one‐quarter of lung cancer patients worldwide have never smoked, particularly among women [54, 55]. In South Asia, 83% of female lung cancer patients have never smoked, whereas in East Asia, the figure is 61% [54]. Similarly, a prospective analysis conducted in Japan indicates comparable incidence rates of lung cancer among never‐smokers and smokers [56]. This emphasizes the vital importance of targeted screening strategies among non‐smokers to improve their participation rates [55].

In addition to the common factors identified across three outcomes, individuals with health insurance were more likely to have greater awareness of LDCT and higher odds of participating in LDCT screening previously, while those with higher incomes had lower odds of greater awareness. The positive association between health insurance in awareness of LDCT and higher odds of LDCT experience may be attributed to the insurance coverage, which facilitates access to healthcare services and increases contact with health professionals, thereby promoting awareness of screening options [57, 58, 59]. Conversely, the negative association between income and the awareness of LDCT contrasts with prior studies in the United States, where higher income was positively associated with willingness to screen [60] and, although not statistically significant, with greater awareness of screening [61]. This discrepancy may be due to cultural or healthcare system differences, warranting further investigation. One possible explanation of that phenomenon may be due to the limited information accessed in the community. Currently, Hong Kong lacks a large‐scale lung cancer screening program and promotional activities; only a few community‐based pilot initiatives and promotions are operated by non‐profit organizations, while the resources from NGO may be more directed toward vulnerable groups [62].

Additionally, we found that although individuals who self‐identified as unhealthy were not significantly associated with willingness to undergo LDCT compared to their counterparts, those who perceived themselves as unhealthy were less likely to have participated in LDCT screening previously. The specific reasons behind the phenomenon remain unclear, but it may be attributed to the amplified perceptions of screening barriers, fear, and resistance of screening, as a study found that perceived barriers were significantly associated with self‐perceived health [63]. Interestingly, our study found that participants with higher perceived barriers were more likely to have received LDCT screening in the past. Numerous studies have confirmed a negative association between perceived barriers and healthy behavior [30, 64, 65]. This discrepancy may be attributed to the influence of past experiences and the inverse relationship. Individuals with prior LDCT screening experience have greater knowledge of the screening process, making them more likely to identify and consider potential barriers, such as financial costs and scheduling conflicts [66]. Furthermore, considering that we collected this data retrospectively, the root cause of this association may stem from past experiences amplifying participants' subjective evaluations.

### 4.3 Implications for Practice and Research

The findings of this study may inform local practice and policy in Hong Kong and similar settings. First, the suboptimal willingness and limited awareness of LDCT screening among high‐risk individuals underscore the urgent need for community‐based LDCT screening education and outreach campaigns that focus primarily on its benefits to raise awareness and promote early lung cancer detection. Tailored health communication strategies should concentrate on enhancing self‐efficacy while addressing misconceptions and emotional barriers to screening. Second, the strong association between cues to action and screening behavior suggests that interventions involving healthcare professionals, family members, and community advocates could play a key role in encouraging participation. Integrating routine risk assessment and screening recommendations into primary care consultations may be beneficial. Third, disparities in screening awareness and uptake by marital status, income, and insurance coverage indicate the need to address social and structural determinants of health. Policymakers should consider expanding subsidized or publicly funded LDCT screening programs, particularly for vulnerable groups who may lack access to private insurance or health information. In addition, developing public‐private partnerships and collaborating with the private sector may help promote access among potential service recipients who are less likely to use local community resources. Lastly, the utilization of the HBM in this study provides a valuable framework for designing behavior modification interventions. These may include enhancing cues to action and self‐efficacy through measures such as reminders and social support to promote lung cancer screening. Further longitudinal and interventional studies are needed to explore causal relationships and evaluate the effectiveness of specific strategies, such as social support interventions, in improving screening uptake.

### 4.4 Strengths and Limitations

This study is one of the few to investigate willingness, awareness, and past uptake of LDCT for lung cancer screening among high‐risk individuals in Hong Kong, and provides valuable insights into associated factors in an East Asian urban context. It adopted a theory‐informed approach, using the HBM to identify psychological constructs associated with lung cancer screening. The use of multivariable regression analyses allowed for adjusting for potential confounders, increasing the robustness of associations observed. Furthermore, the study included a relatively large and diverse sample, which enhances the robustness of the findings.

However, some limitations should be acknowledged. First, as a cross‐sectional study, this research only captured data at a single time point, strictly limiting the ability to establish causal relationships between factors (e.g., HBM constructs) and willingness to undergo LDCT. Our findings can only suggest associations. Longitudinal studies would be valuable in examining how different factors may impact the perceptions and participation of screening over time. Second, the study relied on self‐reported data, which may be subject to recall bias or social desirability bias, especially for sensitive behaviors such as smoking. Third, the findings of this study should be interpreted with caution, as the convenience sampling method may introduce sampling bias and limit generalizability. Lastly, the study did not explore structural barriers such as cost, screening availability, or healthcare system factors in depth, which may also play a critical role in LDCT uptake.

## 5 Conclusion

This study revealed a suboptimal level of willingness of LDCT lung cancer screening among high‐risk individuals in Hong Kong; only 57.3% of participants expressing willingness to receive screening within the next year. Key factors influencing willingness included self‐efficacy, perceived benefits, cues to action, and smoking‐related exposures, while awareness was associated with insurance coverage, smoking status, and family history of lung cancer. Social determinants such as marital status also played important roles. These findings underscore the need for targeted interventions to improve knowledge, confidence, and access to screening, particularly among vulnerable populations.

## Author Contributions

Claire Chenwen Zhong, Junjie Huang, and Martin C.S. Wong conceptualized and supervised the study. Zhaojun Li and Claire Chenwen Zhong were responsible for data curation and formal analysis. Claire Chenwen Zhong, Zehuan Yang, and Mingtao Chen drafted the manuscript. Junjie Huang and Martin C.S. Wong reviewed and revised the manuscript. All authors read and approved the final manuscript.

## Funding

This study was funded by the AstraZeneca Hong Kong Limited.

## Ethics Statement

The Survey and Behavioral Research Ethics Committee of the Chinese University of Hong Kong approved the study (approval no. SBRE‐24‐0083).

## Consent

The authors have nothing to report.

## Conflicts of Interest

The authors declare no conflicts of interest.

## Supporting information


**Table S1:** Summary of key findings.
**Table S2:** VIF test.
**Figure S1:** Forest plot of significant factors across outcomes.

## Data Availability

The datasets used and/or analyzed during the current study are available from the corresponding author on reasonable request.
