# Supplementary material for: Acceptance of Lung Cancer Screening and Associated Factors in Hong Kong: A Population‐Based Study
Source: Cancer Med. 2026 Jan 7;15(1):e71513. doi: 10.1002/cam4.71513 (PMC12778295; doi:10.1002/cam4.71513)

Supplementary Table 1. Summary of Key Findings

|  | Willingness to receive LDCT | | Awareness of LDCT | | Received LDCT | |
| --- | --- | --- | --- | --- | --- | --- |
|  | aOR (95%CI) | P value | aOR (95%CI) | P value | aOR (95%CI) | P value |
| Age Group |  |  |  |  |  |  |
| 45-54 years | Reference |  | Reference |  | Reference |  |
| 55-65 years | 1.081 (0.795-1.469) | 0.620 | 0.900 (0.648-1.248) | 0.527 | 0.838 (0.515-1.363) | 0.476 |
| Over 65 years | 1.900 (1.074-3.362) | **0.027** | 0.504 (0.237-1.073) | 0.075 | 0.434 (0.118-1.598) | 0.210 |
| Marriage |  |  |  |  |  |  |
| Married | Reference |  | Reference |  | Reference |  |
| Unmarried | 0.678 (0.486-0.946) | **0.022** | 0.602 (0.403-0.897) | **0.013** | 0.993 (0.544-1.812) | 0.981 |
| Household income (Monthly) |  |  |  |  |  |  |
| <30,000 | Reference |  | Reference |  | Reference |  |
| 30,000-49,999 | 1.210 (0.836-1.750) | 0.312 | 0.939 (0.630-1.400) | 0.758 | 1.139 (0.628-2.068) | 0.668 |
| ≥50,000 | 0.838 (0.557-1.261) | 0.396 | 0.491 (0.305-0.792) | **0.004** | 0.662 (0.320-1.369) | 0.266 |
| Self-report health |  |  |  |  |  |  |
| Good | Reference |  | Reference |  | Reference |  |
| Not Good | 1.153 (0.848-1.566) | 0.364 | 0.876 (0.626-1.227) | 0.441 | 0.506 (0.307-0.833) | **0.007** |
| Health insurance |  |  |  |  |  |  |
| No | Reference |  | Reference |  | Reference |  |
| Yes | 1.227 (0.908-1.659) | 0.183 | 1.767 (1.253-2.492) | **0.001** | 2.538 (1.441-4.471) | **0.001** |
| Smoking |  |  |  |  |  |  |
| Never Smoker | Reference |  | Reference |  | Reference |  |
| Current/ex-smoker | 1.710 (1.220-2.396) | **0.002** | 1.907 (1.336-2.723) | **<0.001** | 2.003 (1.178-3.403) | **0.010** |
| Secondhand smoke |  |  |  |  |  |  |
| Never | Reference |  | Reference |  | Reference |  |
| Seldom | 1.380 (0.974-1.955) | 0.070 | 1.206 (0.802-1.815) | 0.368 | 1.328 (0.681-2.588) | 0.405 |
| Frequently | 2.099 (1.236-3.566) | **0.006** | 1.634 (0.965-2.768) | 0.068 | 1.979 (0.910-4.304) | 0.085 |
| Cooking at home |  |  |  |  |  |  |
| No | Reference |  | Reference |  | Reference |  |
| Yes | 1.476 (1.082-2.013) | **0.014** | 0.991 (0.708-1.389) | 0.960 | 1.203 (0.727-1.990) | 0.471 |
| Family history of lung cancer |  |  |  |  |  |  |
| No | Reference |  | Reference |  | Reference |  |
| Yes | 1.442 (0.963-2.160) | 0.076 | 1.962 (1.329-2.896) | **0.001** | 2.664 (1.583-4.483) | **<0.001** |
| Perceived benefits |  |  |  |  |  |  |
| Low | Reference |  | Reference |  | Reference |  |
| High | 1.921 (1.428-2.586) | **<0.001** | 1.680 (1.218-2.316) | **0.002** | 1.287 (0.798-2.076) | 0.301 |
| Perceived barriers |  |  |  |  |  |  |
| Low | Reference |  | Reference |  | Reference |  |
| High | 1.263 (0.944-1.689) | 0.116 | 0.927 (0.674-1.273) | 0.638 | 1.831 (1.151-2.913) | **0.011** |
| Self-efficacy |  |  |  |  |  |  |
| Low | Reference |  | Reference |  | Reference |  |
| High | 3.313 (2.461-4.462) | **<0.001** | 1.705 (1.224-2.374) | **0.002** | 1.464 (0.885-2.420) | 0.138 |
| Cue to action |  |  |  |  |  |  |
| Low | Reference |  | Reference |  | Reference |  |
| High | 1.672 (1.252-2.231) | **<0.001** | 2.631 (1.894-3.653) | **<0.001** | 3.859 (2.169-6.863) | **<0.001** |

Supplementary Table 2. VIF Test

1. VIF Test for Factor Association with Willingness to Undergo LDCT Screening

|  | **GVIF** | **Df** | **GVIF^(1/(2*Df))** |
| --- | --- | --- | --- |
| Age_group | 1.410572 | 2 | 1.089805 |
| Sex | 1.239124 | 1 | 1.11316 |
| Marriage | 1.233084 | 1 | 1.110443 |
| Education | 1.256284 | 2 | 1.058698 |
| Career | 1.316348 | 1 | 1.147322 |
| Family_aver_income | 1.456712 | 2 | 1.09861 |
| Self_report_health | 1.22844 | 1 | 1.10835 |
| Health_insurance | 1.15586 | 1 | 1.075109 |
| Chronic_disease | 1.249456 | 1 | 1.117791 |
| Risky_env | 1.113628 | 1 | 1.055286 |
| Smoking | 1.33475 | 1 | 1.155314 |
| Smoker_social | 1.228982 | 2 | 1.052898 |
| Cooking_home | 1.126135 | 1 | 1.061195 |
| FH_Lungcancer | 1.074021 | 1 | 1.03635 |
| Perceived_Benefits | 1.122961 | 1 | 1.059698 |
| Perceived_Barriers | 1.100522 | 1 | 1.049058 |
| Self_Efficacy | 1.14954 | 1 | 1.072166 |
| Cues_to_action | 1.094239 | 1 | 1.046059 |
| Perceived_threat | 1.20984 | 1 | 1.099927 |

1. VIF Test for Factor Association with Awareness of LDCT Screening

|  | **GVIF** | **Df** | **GVIF^(1/(2*Df))** |
| --- | --- | --- | --- |
| Age_group | 1.257144 | 2 | 1.058879 |
| Sex | 1.239006 | 1 | 1.113106 |
| Marriage | 1.228692 | 1 | 1.108464 |
| Education | 1.287592 | 2 | 1.065233 |
| Career | 1.223059 | 1 | 1.10592 |
| Family_aver_income | 1.446592 | 2 | 1.096697 |
| Self_report_health | 1.270784 | 1 | 1.12729 |
| Health_insurance | 1.12803 | 1 | 1.062088 |
| Chronic_disease | 1.258132 | 1 | 1.121665 |
| Risky_env | 1.115951 | 1 | 1.056386 |
| Smoking | 1.418613 | 1 | 1.191056 |
| Smoker_social | 1.267183 | 2 | 1.060987 |
| Cooking_home | 1.090481 | 1 | 1.044261 |
| FH_Lungcancer | 1.120055 | 1 | 1.058326 |
| Perceived_Benefits | 1.158236 | 1 | 1.076214 |
| Perceived_Barriers | 1.12633 | 1 | 1.061287 |
| Self_Efficacy | 1.194227 | 1 | 1.092807 |
| Cues_to_action | 1.114756 | 1 | 1.05582 |
| Perceived_threat | 1.280712 | 1 | 1.131685 |

1. VIF Test for Factor Association with LDCT Screening Experience

|  | **GVIF** | **Df** | **GVIF^(1/(2*Df))** |
| --- | --- | --- | --- |
| Age_group | 1.283193 | 2 | 1.064322 |
| Sex | 1.184805 | 1 | 1.088488 |
| Marriage | 1.296552 | 1 | 1.138663 |
| Education | 1.368026 | 2 | 1.081493 |
| Career | 1.252935 | 1 | 1.119346 |
| Family_aver_income | 1.507889 | 2 | 1.108134 |
| Self_report_health | 1.328299 | 1 | 1.152519 |
| Health_insurance | 1.141973 | 1 | 1.068632 |
| Chronic_disease | 1.301783 | 1 | 1.140957 |
| Risky_env | 1.158073 | 1 | 1.076138 |
| Smoking | 1.4713 | 1 | 1.212971 |
| Smoker_social | 1.308314 | 2 | 1.069493 |
| Cooking_home | 1.087616 | 1 | 1.042888 |
| FH_Lungcancer | 1.211518 | 1 | 1.10069 |
| Perceived_Benefits | 1.208587 | 1 | 1.099357 |
| Perceived_Barriers | 1.133176 | 1 | 1.064507 |
| Self_Efficacy | 1.234594 | 1 | 1.111123 |
| Cues_to_action | 1.108887 | 1 | 1.053037 |
| Perceived_threat | 1.351917 | 1 | 1.162719 |

Supplementary Figure 1. Forest Plot of Significant Factors Across Outcomes


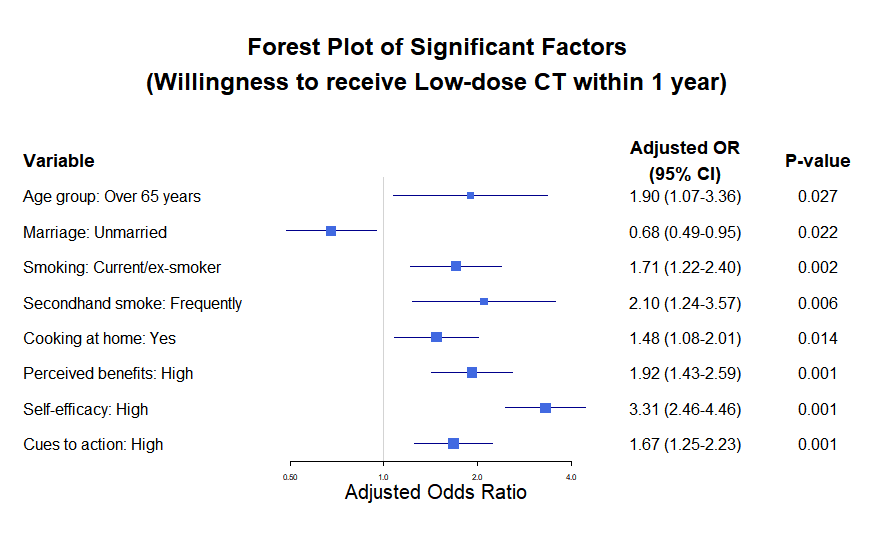


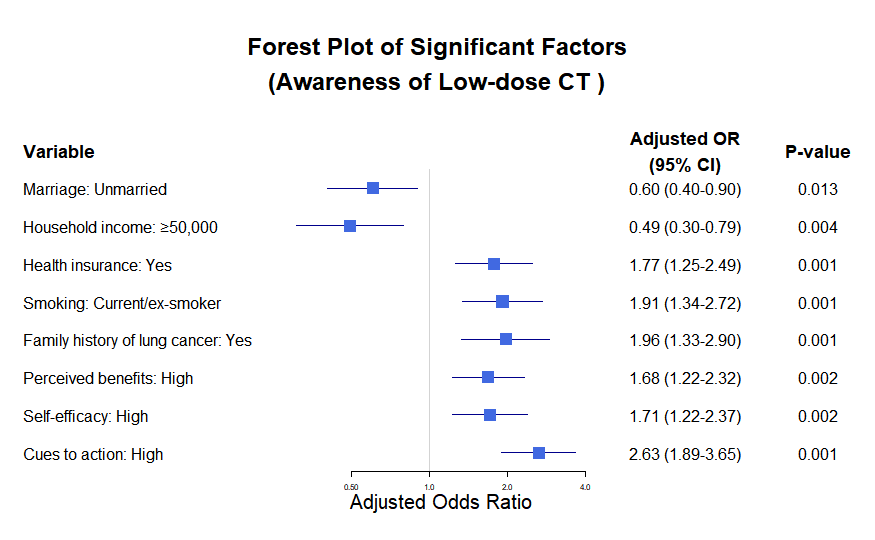


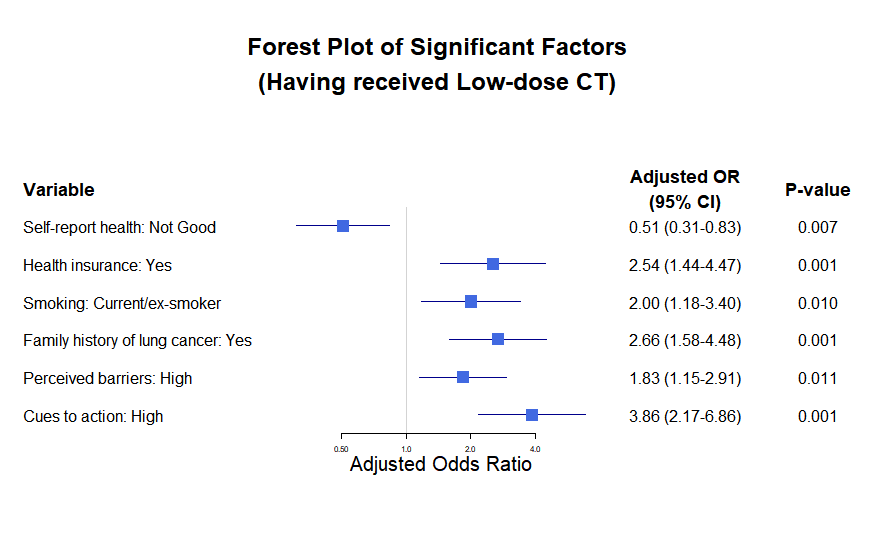

Supplement: Supplementary file 1 — Table S1: Summary of key findings. Table S2: VIF test. Figure S1: Forest plot of significant factors across outcomes. [file CAM4-15-e71513-s001.docx]
